# Water Quality Determination of Küçükçekmece Lake, Turkey by Using Multispectral Satellite Data

**DOI:** 10.1100/tsw.2009.135

**Published:** 2009-11-01

**Authors:** Erhan Alparslan, H. Gonca Coskun, Ugur Alganci

**Affiliations:** ^1^ TUBITAK,Marmara Research Center, Earth and Marine Sciences Institute, 41470 Gebze, Kocaeli, Turkey; ^2^ Istanbul Technical University, Faculty of Civil Engineering, Department of Remote Sensing, 34469 Maslak, Istanbul, Turkey; ^3^ Istanbul Technical University, Center for Satellite Communication and Remote Sensing, 34469 Maslak, Istanbul, Turkey

**Keywords:** multitemporal remote sensing, geographical information systems, water basin, urban monitoring, marine pollution, water quality, multiple regression analysis

## Abstract

This study focuses on the analysis of the Landsat-5 TM + SPOT-Pan (1992), IRS-1C/D LISS + Pan (2000), and Landsat-5 TM (2006) satellite images that reflect the drastic land use/land cover changes in the Küçükçekmece Lake region, Istanbul. Landsat-5 TM satellite data dated 2006 was used for mapping water quality. A multiple regression analysis was carried out between the unitless planetary reflectance values derived from the satellite image and *in situ* water quality parameters chlorophyll a, total phosphorus, total nitrogen, turbidity, and biological and chemical oxygen demand measured at a number of stations homogenously distributed over the lake surface. The results of this study provided valuable information to local administrators on the water quality of Küçükçekmece Lake, which is a large water resource of the Istanbul Metropolitan Area. Results also show that such a methodology structured by use of reflectance values provided from satellite imagery, *in situ* water quality measurements, and basin land use/land cover characteristics obtained from images can serve as a powerful and rapid monitoring tool for the drinking water basins that suffer from rapid urbanization and pollution, all around the world.

## INTRODUCTION

Water quality (WQ) is the general term that describes whether or not the surrounding environment is endangered by pollutants in the water. From a historical perspective, poor WQ was unnoticed before the industrial revolution and the rapid growth of cities. About the turn of the century, WQ was taken under control, concentrating on sewage and industrial discharges[1,2].

The nearness of water reservoirs to cities brings down the cost of supplying water to the users, but also brings in difficulties and problems in the protection and management of the water resources against illegal and unplanned urbanization. The growth of urbanization or changes in land use/land covers (LULC) patterns, which also have adverse impacts on the ecology of the area, especially hydrogeomorphology and vegetation[3]. Since cities need a sustainable water supply, existing water resources and their basins need to be continuously monitored for WQ. The remote sensing (RS) technique has a high potential for such a goal[4,5]. Sensors aboard satellites can measure, at various wavelengths, the amount of solar radiation reflected by surface water, which can be correlated to WQ parameters such as total suspended solids, chlorophyll a concentration, etc.[6].

In order to control and to monitor WQ, routine field studies need to be carried out to protect such vulnerable water bodies. However, classical methods used in making these inventories are rather unsatisfactory compared to modern techniques such as RS and geographical information systems (GIS) in determining and updating information on pollution loads, LULC changes in the watersheds, and WQ[7,8,9,10,11,12].

Istanbul City has the highest population in Turkey, which is 12,249,536 as of 30 November 2007 according to the most comprehensive global news network, The Latest Demography World News (DWN). As a result of a population explosion in recent years, residential areas have expanded beyond the planned urbanization program, which unfortunately also created illegal housing as well. It is obvious that urbanization, either legal or illegal, consumes a vast amount of agricultural land and other green areas, leading to deterioration of the environment. The Istanbul Metropolitan Area has eight surface water reservoirs. Küçükçekmece Lake is one of the most important of these, supplying 120,000 $m^3$/day. It is one of the precious lagoon lakes of the world, having direct connection to the Marmara Sea. Within the last 2 decades, irrigation, urbanization, and other natural conditions have affected the WQ in the Küçükçekmece water reservoir negatively. Pollution loads that have negative effect on the Küçükçekmece Lake water still originate from settlements and industries as point and nonpoint pollutant sources. Polluted rivers and total deposited matter due to erosion also contribute to the pollution in the lake.

In this study, the applied methodology consisted of three steps. In the first step, LULC characteristics of the Küçükçekmece Water Basin were derived from Landsat-5 TM + SPOT-Pan, IRS-1C/D LISS + Pan, and Landsat-5 TM satellite data, respectively, for the years 1992, 2000, and 2006, using a supervised classification technique. In the second step, WQ parameters measured at the lake surface stations were correlated with the spectral reflectance values derived from an atmospherically corrected Landsat-5 TM satellite image of the year 2006 using the multiple regression technique. The spatial distribution of the WQ parameters — chlorophyll a (CHL), total phosphorus (TP), total nitrogen (TN), turbidity (TURB), biological oxygen demand (BOD), and chemical oxygen demand (COD) — were then mapped using the results of these analyses. Finally, the satellite images of the Küçükçekmece water body dated 1992, 2000, and 2006 were classified by a supervised minimum distance classification program in order to investigate temporal changes in lake WQ and to interpret these, taking the LULC classification results into account.

## STUDY AREA

The geographical coordinates of the center of the Küçükçekmece Lake are 28° 45’ East longitude and 41° 00’ North latitude. The location of the Küçükçekmece Water Basin is shown in Fig. 1 within the borders of Istanbul City.

The Küçükçekmece total catchment area is about 340 $km^2$. Küçükçekmece Lake Basin is 15 km away from the west of the city center and it is situated between the west border of Küçükçekmece district and the east border of Avcilar district. The reservoir area of the dam is 15.22 $km^2$. The town of Küçükçekmece has been experiencing rapid urbanization, as can be seen in Table 1, with a high rate of migration during the last 2 decades[13]. Most of the urbanization around the lake was unplanned. The population of the town was 707 in the 1930s and since then, it has increased considerably, reaching to 350,000 in the 1990s and 600,000 in the year 2000. Therefore, the lagoon has been subjected to heavy

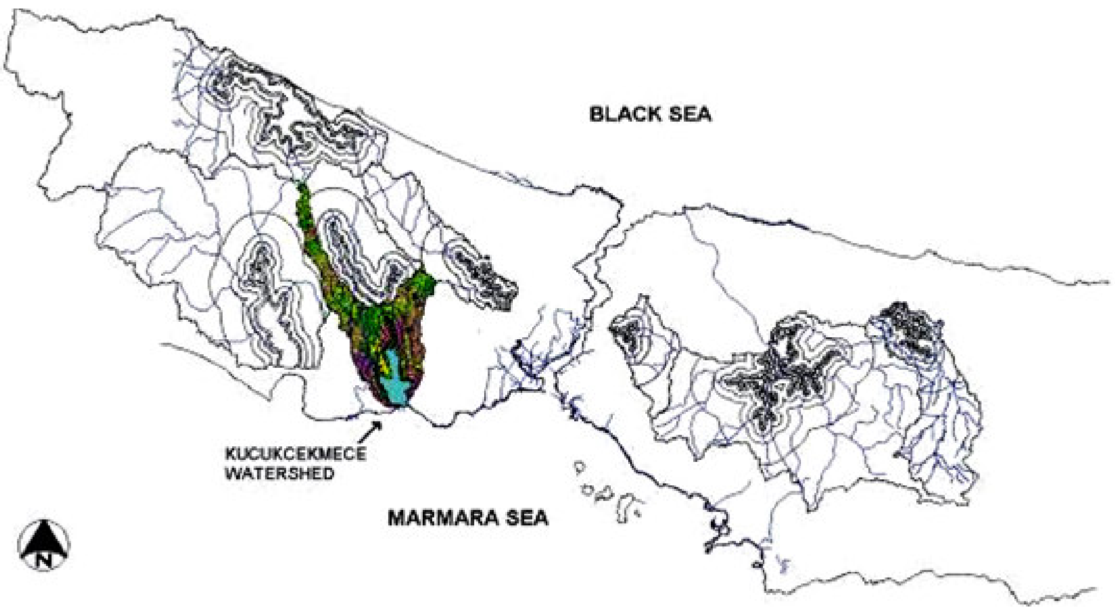

**FIGURE 1**. Location of Küçükçekmece Water Basin in Istanbul Province.

**TABLE 1**
**Temporal Urban Area Change in Küçükçekmece Water Basin during 1992–2006**

| Küçükçekmece | Urban (U) (ha) | Total Basin Area (TBA) (ha) | % U/TBA |
|---|---|---|---|
| 1992 | 728.85 | 16,308.77 | 4.47 |
| 2000 | 989.021 | 16,308.77 | 6.06 |
| 2006 | 3,626.980 | 16,308.77 | 22.24 |

nutrient inputs because of the poor sanitary treatment of wastewater associated with human population growth around the lake. In addition, a considerable amount of industrial waste has been introduced directly into the lagoon.

## METHODOLOGY

### Image Processing

During the analyses of urban changes in the Küçükçekmece Water Basin, Landsat-5 TM + SPOT-Pan, IRS-1C/D LISS + Pan, and Landsat-5 TM satellite data were used, respectively, for the years 1992, 2000, and 2006. The spatial resolutions for SPOT-XS and Pan, IRS1-C/D LISS and Pan, and Landsat-5 TM are, respectively, 20 and 10 m, 23.5 and 5.8 m, and 30 m.

In this study, both 1:25,000 and 1:5,000 scaled digital maps that cover the study area and orthophotos derived from 1:5000 aerial photographs were used as ground truth information in rectification of the satellite imagery.

The satellite imagery data were transformed into the UTM International 1909 Zone 35 coordinate system using the 1:5,000 digital topographic maps in order to achieve the necessary geometric registration. Images are geometrically corrected with 50 ground control points selected from maps and images with registration accuracy about ±0.5 pixels root mean square error (RMSE). Radiometric image enhancement techniques, such as contrast enhancement and histogram matching, were then applied for all rectified satellite images band by band. After applying these necessary image enhancements needed for data fusion, the IHS transform-based data fusion method was used to obtain merged, multisensor images (TM + SPOT-Pan, IRS LISS + Pan) where the color information in the multispectral image and the spatial resolution of the panchromatic image were preserved[14] (Fig. 2).

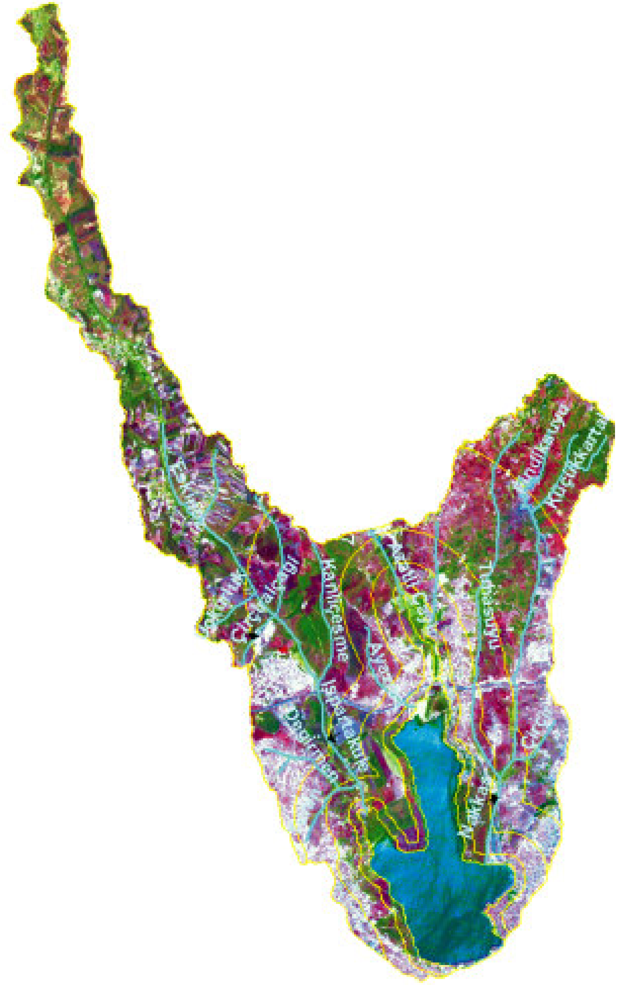

**FIGURE 2**. A Landsat-5 TM + SPOT-Pan color composite satellite image of Küçükçekmece Water Basin dated 1992. Streams and protection zones were overlaid as vector data, applying GIS software.

## Land Use Classification

There are two primary methods of image classification utilized by image analysts, namely, unsupervised and supervised classification. The unsupervised image classification technique classifies the pixels without any input class information from the user, according to the clusters formed in an n-dimensional pattern space, provided each dimension corresponds to a particular spectral band of the satellite image. On the other hand, supervised image classification requires the assistance of an analyst to define small test areas, called training sites, on the image to represent a desired land cover category. After a signature for each land cover category has been defined, the classification software uses them to classify the remaining pixels[15,16].

In this study, a supervised classification technique called the Maximum Likelihood (Bayesian) decision rule was used for LULC classification. New samples from training sites were selected according to the ground truth data and field works. Totally, 400 training sites were selected, then added to a signature file to form 80 different classes. In the next step, these 80 classes were reduced in number to ten final LULC classes by recode operation. These ten classes were verified on the computer display screen by confirmation through the ground samples taken from the experimental area with accurate field information. As the current study was performed much later than the year 2000, it was difficult to obtain the past residential and forest ground truth information while classifying 1992 and 2000 dated images. These difficulties were eliminated, especially in residential and forest areas, by using 1996 dated orthophotos and getting samples of the known structures from the preceding classified images. Also, previously dated forest stance maps were used for training site selection for these two dates. Resultant classified images are shown in Fig. 3 for the studied years. The classification results were assessed for accuracy[17], selecting 100 random pixels in the classified images for three different years and comparing them with ground truth data. In this Küçükçekmece study, the overall accuracy was found to be 82, 81, and 82% with 0.78, 0.74, and 0.76 kappa results, respectively, for the years 1992, 2000, and 2006 for randomly selected 100 points. The kappa statistics show the degree of agreement between Producers Accuracy and Users Accuracy in the Classification Accuracy assessment process. Values near to 1 express higher agreement degree.

## Reflectance Conversion and Regression Analysis

When reflectance characteristics of specific surfaces are studied, using raw digital numbers (DN) in satellite images introduce errors due to noise and value offsets in addition to spectral reflectance. These are the effects of the viewing geometry of the satellite, the angle of the sun's incoming radiation, atmospheric depth due to viewing angle, and the design characteristics of the sensor. As the reflectance values of Küçükçekmece Lake surface and WQ parameters measured at station locations shown in Fig. 4 would be correlated with multiple regression analysis, DN first needed to be converted into reflectance values. For this aim, multispectral bands of July 2006 Landsat-5 TM data were used.

Conversion was done in two steps[18]. The first step was the conversion of DN to radiance, which removed the voltage bias and gains from the satellite sensor. At the second step, the radiance values were converted into at-satellite reflectance values, taking into account the varying sun angles due to differences in latitude, season, and time of day, and the variation in the distance between the Earth and sun.

Afterwards, a further atmospheric correction became necessary in order to obtain correct results, because electromagnetic energy radiated by objects exhibits different properties under different atmospheric conditions dependent on the illumination and the geometrical shape of the object, mist, fog, and haze in the air[19]. The atmospheric correction was done using the Dark Object Subtraction (DOS) haze reduction method[20]. Therefore, the minimum reflectance value in the infrared band that was measured to be 0.015 was subtracted from the pixel values in all bands. For this particular study area of lake surfaces, even this elementary operation improved the results obtained.

Multiple regression models are widely used, practical, effective, and fast for correlation and estimation of WQ parameters over large areas especially, where *in situ* measurements are limited compared to the spatial coverage of the satellite image. There are several studies available in the literature that present the high performance and efficiency of multiple regression for estimation of WQ with satellite imagery[21,22,23,24]. Chen et al. constructed a GEGA model by incorporating grammatical evolution (GE)

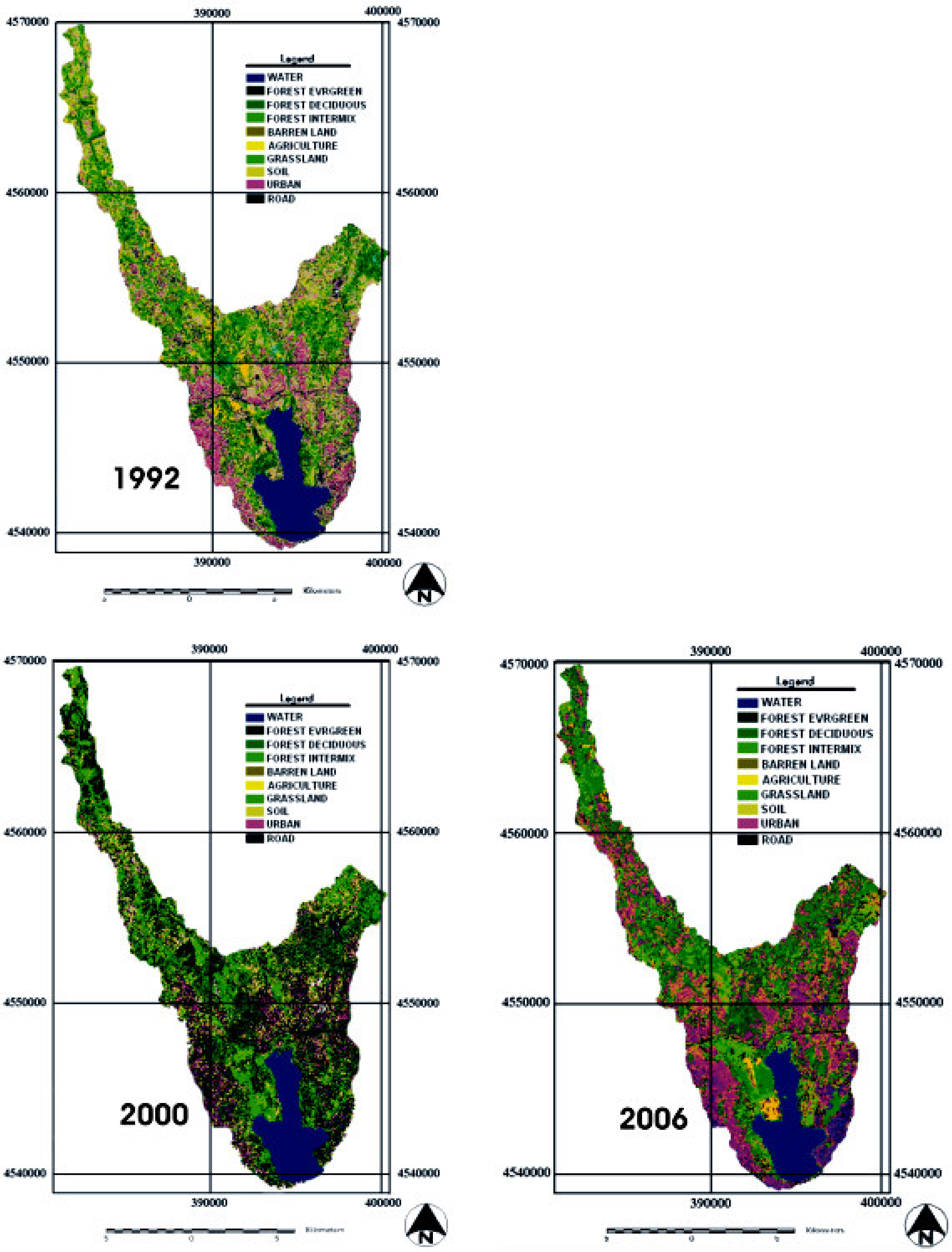

**FIGURE 3**. Classified images of the Küçükçekmece Water Basin dated 1992, 2000, and 2006.

into the parallel genetic algorithm (GA)[25]. When compared with a traditional linear multiple regression, the performance of parallel GEGA was found to be better than that of the multiple regression model, with slightly higher correlation coefficient and slightly lower estimating errors in this study. However, these

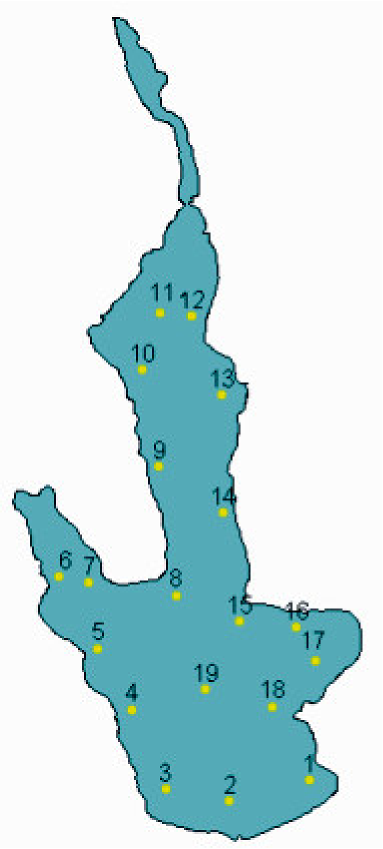

**FIGURE 4**. WQ measurement station locations in the Küçükçekmece Lake.

small differences can be regarded as negligible, considering the complexity and time-consuming structure of the given GEGA model.

In light of this information, a linear multiple regression model was used in this study. WQ parameters measured at each station near time with the satellite image are given in Table 2. Data of eight stations were separated to be used for model testing and the remaining 11 stations were used for construction and calibration of the regression model. Using Tables 2 and 3, a multiple regression analysis was carried out to determine the model coefficients, a–g, to calculate WQ parameter at any location on the lake according to Eq. 1.

$$WQ = a + b*x_1 + c*x_2 + d*x_3 + e*x_4 + f*x_5 + g*x_6 \quad (1)$$

where $x_k$, unitless planetary reflectance values at different satellite image bands, $1 \leq k \leq 7$; a–g, coefficients for the model.

These coefficients and multiple R values for each WQ parameter are listed in Table 4, which reveals that the highest multiple R values were obtained for TN and TURB, which give the most accurate results. The next best set of multiple R values are for TP and COD, both near to 0.8. However, multiple R values for BOD and CHL are, respectively, 0.58 and 0.52, which show that moderate results will be obtained for these two WQ parameters. These results show that analysis of some of the WQ parameters can be achieved by use of reflectance information derived from satellite imagery.

**TABLE 2**
**WQ Parameters Measured at Stations**

| Station No. | CHL ($mg/m^3$) | BOD (mg/l) | COD (mg/l) | TN (mg/l) | TP (µg/l) | TURB (NTU) |
|---|---|---|---|---|---|---|
| 1 | 1.87 | 30.00 | 51.00 | 0.56 | 2.01 | 7.54 |
| 2 | 1.63 | 48.00 | 61.00 | 1.12 | 0.62 | 7.82 |
| 3 | 2.74 | 46.00 | 58.00 | 0.56 | 1.49 | 9.60 |
| 4 | 2.00 | 46.00 | 69.00 | 0.56 | 1.49 | 8.40 |
| 5 | 3.99 | 38.00 | 62.00 | 1.12 | 2.00 | 9.01 |
| 6 | 0.62 | 64.00 | 113.00 | 17.92 | 5.78 | 33.50 |
| 7 | 2.07 | 26.00 | 80.00 | 0.56 | 1.57 | 7.02 |
| 8 | 2.42 | 12.00 | 83.00 | 0.56 | 2.18 | 11.20 |
| 9 | 1.12 | 30.00 | 70.00 | 0.56 | 1.56 | 8.57 |
| 10 | 0.70 | 32.00 | 63.00 | 0.56 | 2.02 | 8.20 |
| 11 | 1.39 | 14.00 | 60.00 | 0.56 | 1.56 | 4.51 |
| 12 | 2.92 | 14.00 | 45.00 | 0.56 | 1.04 | 2.90 |
| 13 | 2.47 | 22.00 | 76.00 | 0.56 | 1.47 | 8.38 |
| 14 | 0.34 | 26.00 | 80.00 | 0.56 | 2.49 | 9.16 |
| 15 | 0.96 | 30.00 | 66.00 | 0.56 | 2.16 | 9.07 |
| 16 | 1.70 | 28.00 | 72.00 | 11.76 | 4.49 | 28.00 |
| 17 | 1.73 | 22.00 | 70.00 | 0.56 | 1.99 | 9.49 |
| 18 | 3.76 | 32.00 | 72.00 | 0.56 | 2.48 | 7.05 |
| 19 | 0.82 | 20.00 | 66.00 | 0.56 | 1.40 | 6.75 |

The results of the multiple regression analysis are then tested comparing observed WQ parameters vs. predicted WQ parameters from the reflectance values. This comparison is graphically given for all parameters in Fig. 5. Finally, the WQ lake surface spatial distribution maps are created as shown in Fig. 6. These distribution maps illustrate the visual results of a geostatistically based model constructed with WQ parameters and satellite reflectance values.

## WQ Classification

As the regression results demonstrated a strong relationship with reflectance values, 1992 Landsat-5 TM + SPOT-Pan, 2000 IRS-1C/D LISS + Pan, and 2006 Landsat-5 TM satellite images of the Küçükçekmece Lake were classified by a minimum distance supervised classification process in order to obtain final WQ images shown in Fig. 7. Training sites were selected according to station points where the WQ parameters were measured. Also, information about rivers that pour their water into the lake was taken into consideration. The parts of the lake are represented in red, yellow, green, and blue colors, where the creeks empty, which correspond to different water characteristics below:

- Yellow: nutrient pollution–loaded agricultural wastewater
- Green: phosphorous pollution–loaded water, which comes from forest areas
- Red: total suspended sediment pollution loaded with a mixture of the urbanization, agriculture, and industry wastewater discharges
- Blue: natural lake water

**TABLE 3**
**Landsat-5 TM Satellite Image Reflectance Values at WQ Measurement Stations**

| Station No | Band 1 | Band 2 | Band 3 | Band 4 | Band 5 | Band 7 |
|---|---|---|---|---|---|---|
| 1* | 0.0905 | 0.0582 | 0.0437 | 0.0367 | 0.0106 | 0.0084 |
| 2 | 0.0935 | 0.0612 | 0.0488 | 0.0334 | 0.0127 | 0.0116 |
| 3* | 0.0935 | 0.0612 | 0.0488 | 0.0367 | 0.0106 | 0.0084 |
| 4 | 0.0860 | 0.0582 | 0.0437 | 0.0367 | 0.0127 | 0.0084 |
| 5* | 0.0950 | 0.0610 | 0.0488 | 0.0367 | 0.0127 | 0.0084 |
| 6 | 0.0980 | 0.0643 | 0.0540 | 0.0497 | 0.017 | 0.0116 |
| 7 | 0.0905 | 0.0582 | 0.0463 | 0.0334 | 0.0127 | 0.0084 |
| 8* | 0.0860 | 0.0582 | 0.0463 | 0.0367 | 0.0127 | 0.0053 |
| 9 | 0.0920 | 0.0643 | 0.0488 | 0.0400 | 0.0149 | 0.0084 |
| 10 | 0.0890 | 0.0612 | 0.0437 | 0.0367 | 0.0062 | 0.0053 |
| 11 | 0.0935 | 0.0643 | 0.0514 | 0.0367 | 0.0127 | 0.0147 |
| 12* | 0.0935 | 0.0612 | 0.0437 | 0.0302 | 0.0062 | 0.0022 |
| 13 | 0.0920 | 0.0582 | 0.0488 | 0.0400 | 0.0127 | 0.0084 |
| 14* | 0.0920 | 0.0582 | 0.0437 | 0.0334 | 0.0127 | 0.0053 |
| 15 | 0.0920 | 0.0582 | 0.0463 | 0.0334 | 0.0106 | 0.0053 |
| 16* | 0.0935 | 0.0643 | 0.0437 | 0.0367 | 0.0127 | 0.0084 |
| 17 | 0.0905 | 0.0551 | 0.0488 | 0.0367 | 0.0127 | 0.0084 |
| 18* | 0.0890 | 0.0612 | 0.0463 | 0.0334 | 0.0062 | 0.0022 |
| 19 | 0.0905 | 0.0582 | 0.0463 | 0.0334 | 0.0084 | 0.0116 |

* Stations selected as test data locations.

**TABLE 4**
**Coefficients and Multiple R Values Determined for Selected WQ Parameters through Multiple Regression Analysis**

| | a | b | c | d | e | f | g | Multiple R |
|---|---|---|---|---|---|---|---|---|
| TN | –75.621 | 695.301 | 268.014 | –698.023 | 808.053 | 121.984 | –12.783 | 0.835 |
| TP | –12.971 | 137.434 | 39.426 | –176.854 | 236.469 | 40.229 | –82.378 | 0.788 |
| TURB | –85.009 | 877.806 | 315.814 | –1252.402 | 1360.668 | 580.674 | –180.040 | 0.822 |
| BOD | –111.376 | 1186.559 | 1.240 | –618.215 | 1709.467 | –46.207 | 69.027 | 0.576 |
| CHL | –0.637 | –48.729 | 37.731 | 201.612 | –92.556 | –14.391 | –157.944 | 0.513 |
| COD | 31.403 | –610.946 | –422.226 | 871.011 | 1855.762 | 2027.706 | –1544.611 | 0.779 |

Five main creeks that feed the reservoir can be seen clearly in Fig. 7, each carrying some substances to the lake, the location of which can be determined easily. Each of the creeks includes different substances depending on the land use activity of the surrounding area; i.e., loading it with total suspended sediments, residential fall out, nutrients, phosphorus, and others. When a creek passes through a forested area, for instance, the phosphorus load will show a higher concentration than other parameters present in the surface water. Particularly when the locations in red color are investigated through field survey and local data, an increase is observed in urbanization and industrialization in the vicinity of the creek branches

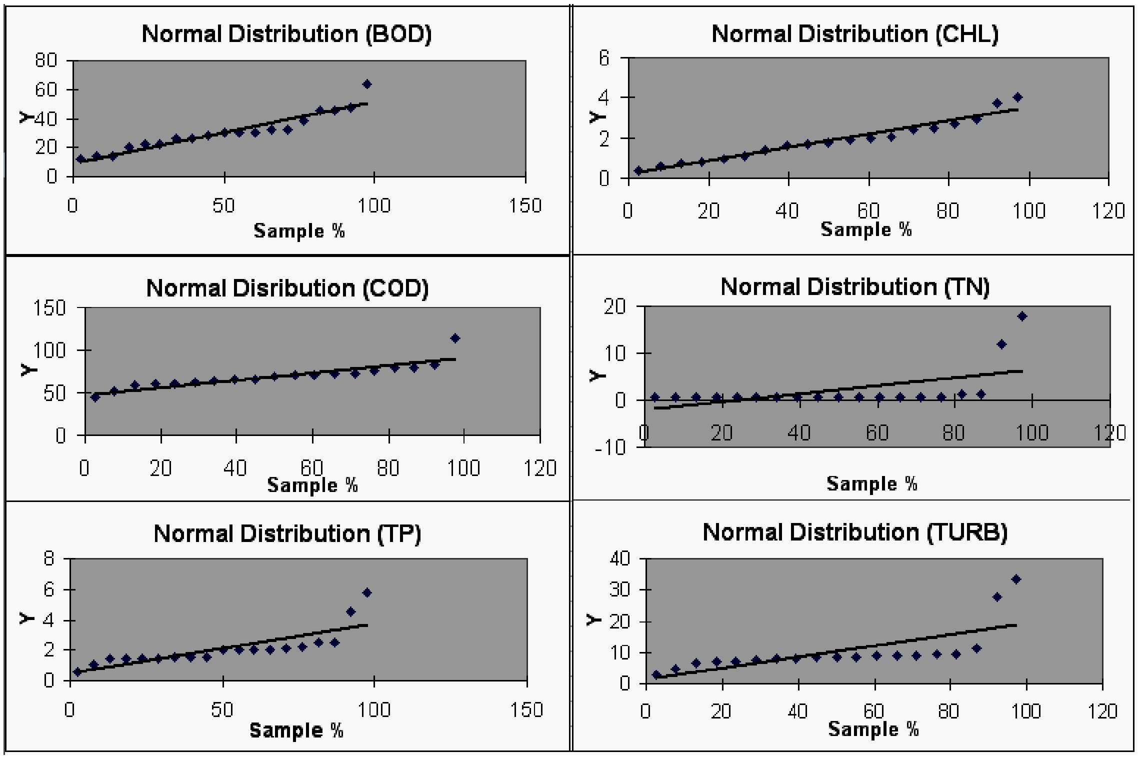

**FIGURE 5**. Normal distribution vs. sample % plots for selected WQ parameters.

that dump into the lake, which carry point source domestic and industrial discharges[5,26]. Analysis of satellite images of the year 2000 reveals regions of different WQ. The Istanbul Water and Sewerage Administration (ISKI) completed the construction of a discharge purification facility in the year 2003 in this region. The discharges previously dumped into the Küçükçekmece Lake and the coasts of the Marmara Sea between Ambarli and Florya are now collected in the Küçükçekmece Discharge Collection Facility and purified by the Southern Küçükçekmece Advanced Biologic Purification Facility and dumped into the Marmara Sea bottom without harming the environment. Thus, the water pollution at the Küçükçekmece Lake and the Marmara Sea is mostly under control. This situation can be seen clearly in the classified 2006 satellite image, where the lake WQ is close to its natural status.

## RESULTS AND DISCUSSIONS

The protection zones around the drinking water resources were determined according to the Water Pollution Control Code (WPCC) that restricts residential, industrial, and agricultural activities in the catchments areas. The WPCC indicates activities that are permissible in each zone: the absolute, short-, medium-, and long-range protection zones[27].

Monitoring environmental protection areas of the water basins by urban planners implies the collection of updated systematic information on LULC patterns and infrastructure facilities. Periodically repeated total field inventories are necessary in order to protect the drinking water dam catchments areas against heavy pollution.

It was shown that changes in LULC in the Küçükçekmece Water Basin for the years 1992, 2000, and 2006 can be identified with this methodology. As seen in Fig. 8, there was an increase in urban areas between these years.

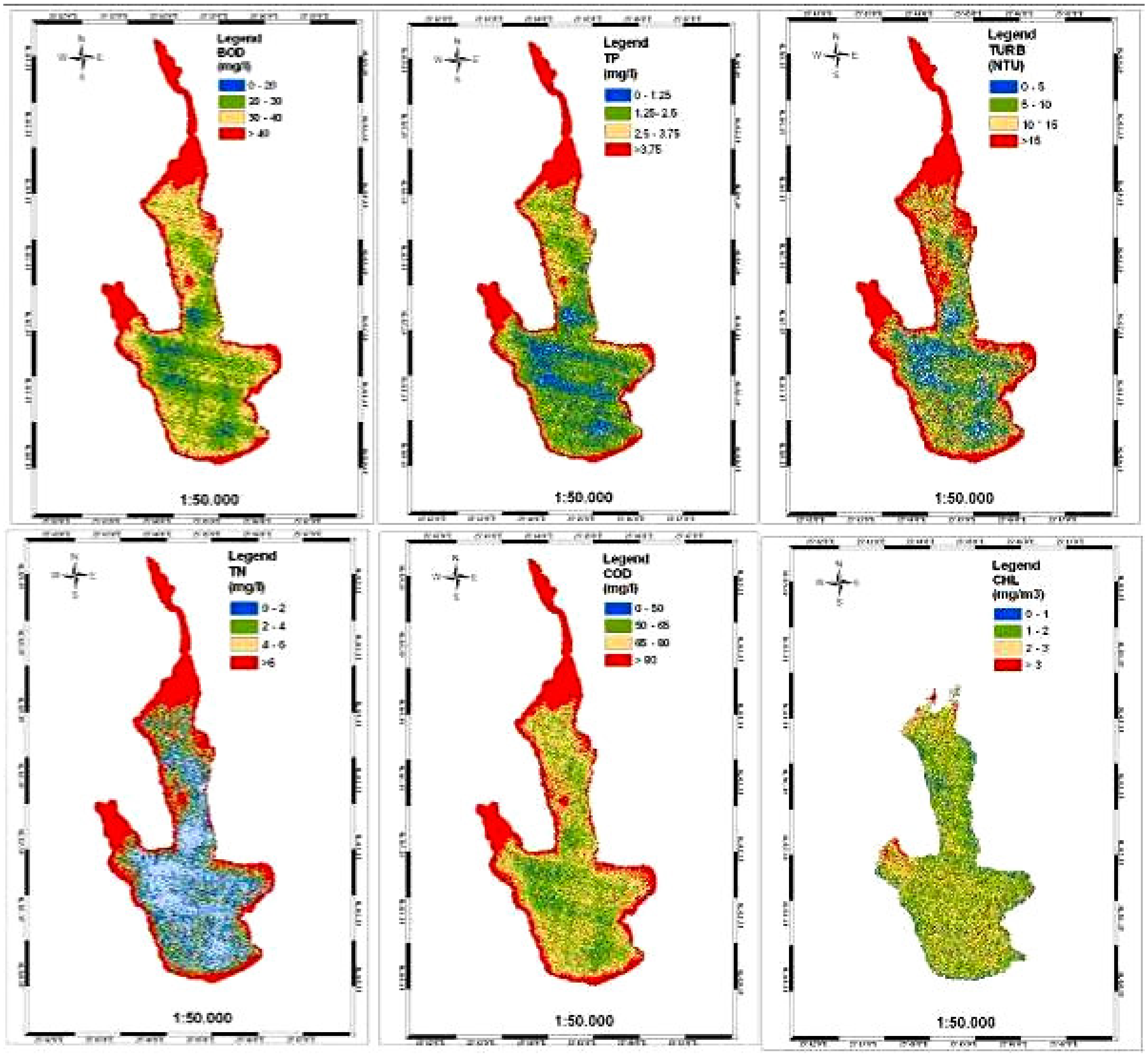

**FIGURE 6**. Selected WQ parameters at Küçükçekmece Lake.

The uncontrolled expansion in urbanization, especially at the southern part of the basin, and motorway construction at the northern part of the basin has caused changes in the shoreline. The urban area increased by 260,171 ha between 1992 and 2000. This difference equals to a 35.7% increase in 8 years. However, the increase of 2,637.96 ha in urban areas between the years 2000 and 2006 gives a dramatic augmentation of 266.72% of the total urban area in 6 years. Despite the negative effects of the 1999 earthquake, there has been an extreme increase in urban areas in this 14-year period[28]. This study shows that remotely sensed data combined with ground truth data makes it possible to explore the LULC management problems associated with the future rapid growth of the Küçükçekmece population.

The main pollutant sources of Küçükçekmece Lake are domestic discharges dumped directly into creeks, industrial discharges, and irrigation water remains enriched in nitrogen and phosphorous compounds swept away from agricultural areas in the hydrologic watershed and precipitation carrying erosion soil. With the Küçükçekmece Environmental Protection Project, ISKI has taken under control the wastewaters polluting the Küçükçekmece Watershed. Owing to this project, wastewater, first flowing directly to the Marmara Sea from Yeşilköy until Ambarlı, is now accumulated by collectors, then transferred into a

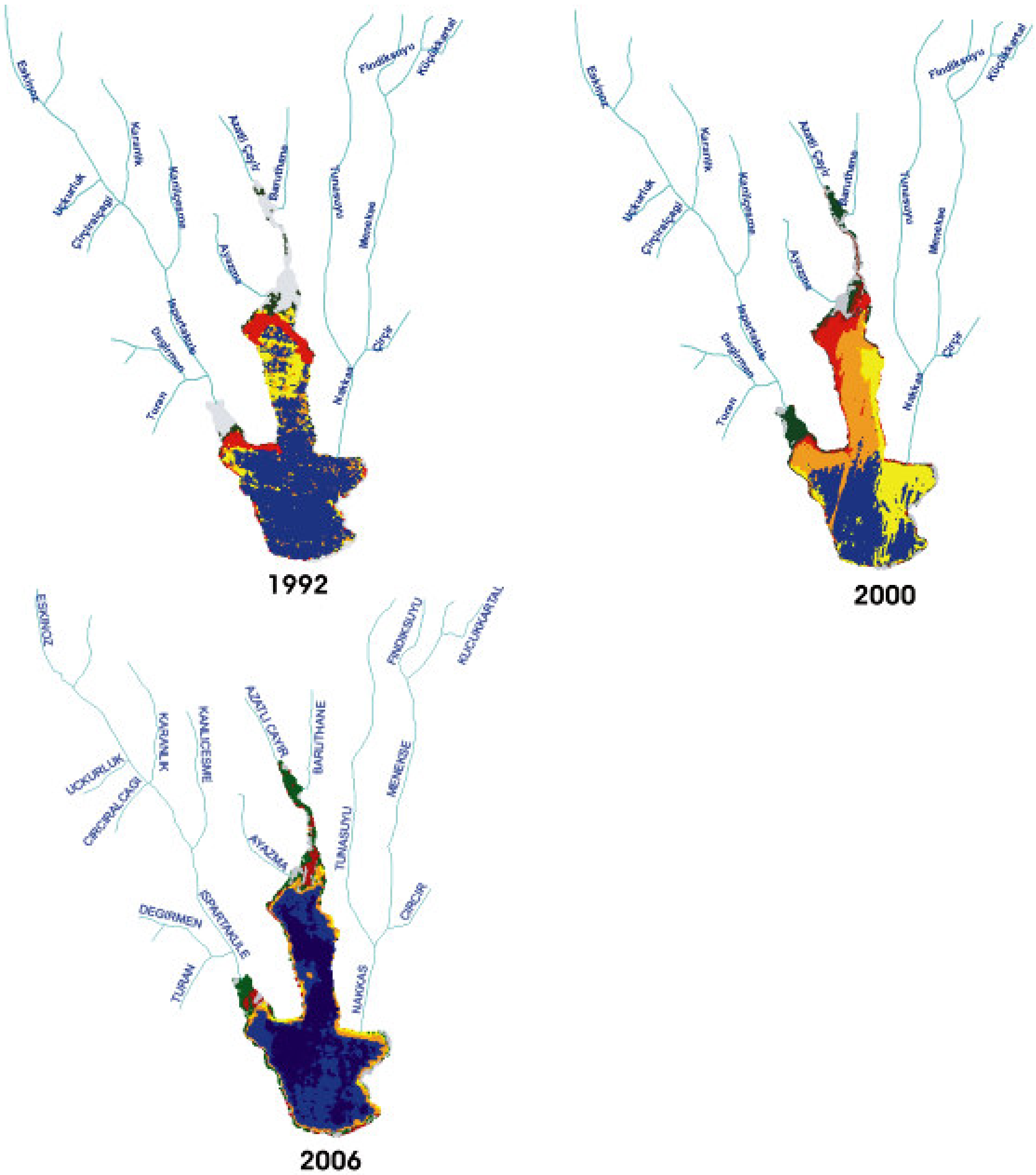

**FIGURE 7**. WQ classified images of Küçükçekmece Lake for 1992, 2000, and 2006.

wastewater precleaning facility, and the coastal zone is mostly liberated from wastewater pollution. Halkalı creek, passing through the basin, but not pouring directly into the body of the lake, but to the sea (not shown in Fig. 7), and Nakkaş creeks are rehabilitated within the framework of the project and their wastewaters that pollute the Küçükçekmece Lake have also been accumulated by collectors to be treated by purifying facilities. Bidding by ISKI still continues to prevent wastewaters flowing into the Küçükçekmece Lake by Sazlıdere creek (with its two main branches named Azatlı Çayı and Baruthane) and Eskinoz creek. Affects of pollutant sources over the lake body are determined with a combined methodology using RS data. Multispectral images are analyzed both with regression analysis and classification methods. Resultant maps

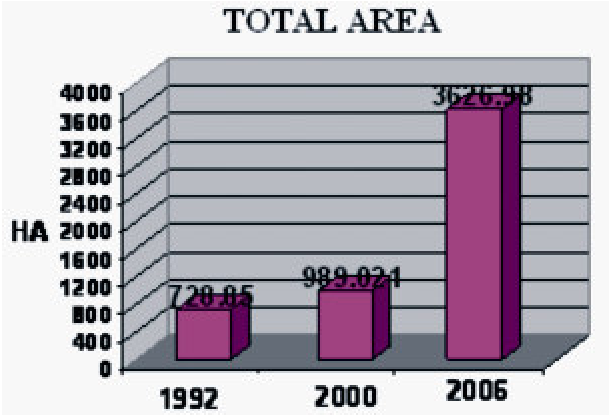

**FIGURE 8.** Three-dimensional temporal urban area change graphics for Küçükçekmece Water Basin.

are produced for both methods. The mentioned WQ situation of the lake can be clearly observed from these maps.

## CONCLUSIONS

Uncontrolled population growth and the rapid development of worldwide urbanization have an extreme impact on the environment. Especially in developing countries, uncontrolled urbanization near water supplies and discharges from industrial activities account for this impact. To minimize the negative impacts to the environment effectively, notably water resources for this study, and to make a control mechanism for population growth, there is a need for accurate, reliable, and up-to-date data at regular intervals. This study introduces an important output for monitoring and evaluating these negative effects in a rapidly urbanizing and industrializing country such as Turkey.

This study shows how RS and GIS techniques are important tools for determining and updating LULC changes at water basin areas, and how WQ measurements at station points can be correlated with spectral reflectance values derived from satellite imagery via regression analysis. In this study, unitless planetary reflectance values of satellite images were used instead of digital numbers for atmospheric correction purposes, while carrying out the regression analysis in determination of WQ parameters. Spatial distribution maps were then created as a geostatistical analysis, in order to illustrate the spatial distribution of WQ parameters individually, which were correlated and tested with spectral reflectance. Moreover, a classification process was applied to satellite images of the lake body. In this process, the station locations where the results of regression gave high correlation with image data and hydrologic ground truth data of rivers, combined with LULC classification, were used in determining sampling locations.

Within this context, this study presents that investigation of WQ with conjunction of LULC analysis is possible with RS images. As large regions of the environment can be studied with satellite imagery, pollution problems can be determined in a fast and precise way. Also, good correlation of observed satellite reflectance with WQ parameters shows that RS data provide a useful index for WQ parameters. If homogenously distributed terrestrial WQ sample measurements will be performed simultaneously with image data acquisition, the described methodology can be performed efficiently in the basins suffering from heavy industrial activities and rapid urbanization all over the world. Moreover, classification of multispectral satellite imagery provides beneficial information for updating the LULC activities in the basin. This valuable information is used to understand the local or total WQ changes, or pollution of the lake body in terms of being domestic/industrial or point or nonpoint pollutants, with support of ground truth information.

To conclude, land use plans should be prepared in accordance with a protection strategy. Local governments, relevant administrations, municipalities, and planning and environmental protection agencies must protect the catchment areas for protection of precious reservoirs of potable water. They must cooperate with universities and scientific organizations, under good coordination, rather than to attempt isolated solutions on an individual basis. Adaptation of the protection strategy to the current status requires certain, urgent, and short-term measures. Future generations can only inherit potable water resources like the Küçükçekmece Lake if and only if land use activities, which can result in irreversible changes, can be kept under control.
